# Suspension in the Treatment of Affections of the Spinal Cord

**Published:** 1889-08

**Authors:** Alexander B. Shaw

**Affiliations:** St. Louis, Mo.; Professor of Diseases of the Mind and Nervous System and Electro-Therapeutics, Beaumont Medical College; Consulting Neurologist, St. Louis Insane Asylum, Alexian Brothers Hospital, St. Louis Railroad Hospital, Etc.


### SUSPENSION IN THE TREATMENT OF AFFECTIONS OF THE SPINAL CORD.[1]

BY ALEXANDER B. SHAW, M .D.,

Professor of Diseases of the Mind and Nervous System and Electro-Therapeutics, Beaumont Medical College; Consulting Neurologist, St. Louis Insane Asylum, Alexian Brothers Hospital, St. Louis Railroad Hospital, Etc.

Suspension in cord affections occurring secondarily to disease of the vertebræ was advocated, and practiced with good results, so long as 1829, by Prof. J. K. Mitchell, of the Jefferson Medical

(1) Read before the St. Louis Medical Society, June 15th, 1889.

College, and has been employed for a number of years by Dr. S. Weir Mitchell[2] in the same class of cases. The latter, indeed, about the time of Motchoukowsky's discovery, was induced to attempt suspension in two cases of spastic paraplegia in which no spinal caries existed; the excellent results obtained from it in pressure paralysis having led him to believe that stretching the cord, and thus altering its vascular conditions, even when signs of meningeal inflammation were absent, would helpfully affect both cord and membranes. Unfortunately, in these cases, no benefit followed; had it been otherwise, suspension would, no doubt, long since have been generally recognized as a useful adjunct to our scanty therapy in chronic degenerations of the spinal cord.

The fact that suspension is beneficial in the treatment of locomotor ataxia, was discovered by Dr. Motchoukowsky, a Russian physician, in 1883, while treating a patient for spinal curvature, who was then suffering from ataxic symptoms. He observed that amelioration of the ataxia followed the suspensions made during the applications of the plaster jacket used for the relief of the curvature, and subsequently suspended other ataxic patients with like favorable results. This important discovery was chronicled and published in a *brochure* on the subject by Dr. Motchoukowsky, in 1883, in which considerable improvement was ascribed to it in twelve tabetic persons; also in various neurasthenias, independent of tabes, in which the sexual functions were reëstablished by this treatment. But, until quite recently, it has been almost barren of results.

However, on the 15th of last January, Prof. Charcot, of Paris, delivered a clinical lecture at the Salpêtrière, in which he stated that he had recently been experimenting with the suspension treatment of locomotor ataxia, and had been obtaining very remarkable results. Charcot's lecture was published in *Le Progrès Médical*, January 19 and February 23, 1889, in which is graphically described the technical details of suspension, as practiced by him, with an ordinary Sayre's suspension apparatus.

(2) *Vide* Treatment of Pott's Paralysis by Suspension, etc., by S. Weir Mitchell, M. D., American Journal of the Medical Sciences, May, 1889.

After describing the apparatus, he lays great stress on the danger of causing unpleasant head symptoms, from compression of the blood-vessels in the neck, if the straps are not properly adjusted. Further on, he says: "When the head is duly disposed of,[3] the shoulder-pieces are slipped under the armpits. Though they may appear of minor importance, they really play the part of regulators during the period of suspension. *For*[4] *it is necessary that, whilst lifted off the ground, the patient should not be entirely supported by the head-piece, for then the traction would become, in some cases, at least, absolutely intolerable.* * * * Careful trials are necessary to determine the exact length of the several straps. * * * While suspended, the patient is made, at intervals of fifteen or twenty seconds, to raise the arms laterally away from the body, so as to transfer more weight upon the head-piece, and so render the traction upon and elongation of the vertebral column still more complete—as complete as is tolerated by each individual. * * * As a rule, the longest time of suspension must not go beyond four minutes, three minutes being taken as the average. Half of a minute is enough at the outset, the maximum being reached during the first six or eight applications of treatment. It is essential to take into account certain individual susceptibilities or physical peculiarities, among which stands foremost the body weight; for, whilst a person of from about 130 to 150 pounds may be suspended forthwith during two minutes or more, the case is quite different in those whose weight reaches 180 pounds or more. In the latter, the tension to which the structures of the neck are subjected may become very severe and painful, and be felt sometimes for a whole day afterwards. * * * It is well to note that certain patients have such a wish—a very natural wish—to get better, that they think themselves bound to stand any amount of pain without complaining; but this circumstance is positively detrimental to the success of treatment, which must be accompanied with but trifling discomfort, at the most, without real pain or fatigue, lest it should defeat its own ends. * * * Suspension must not be carried out

(3) A. de Watteville, British Med. Jour., March 9, 1889.

(4) Italics ours.

oftener than once on alternate days, otherwise it may become more hurtful than beneficial. * * * When the full time has elapsed, the operator very gradually lets the rope loose, so as to avoid *every trace*[5] *of jerking* during the descent. The patient is to be supported while being freed from the apparatus, and made to rest awhile in an arm-chair, brought near for the purpose."

Up to April 1st, Charcot reports over 800 suspensions, with varying degrees of success, but, in the vast majority, walking is improved after the first suspension.[6] At first, this improvement lasts but a few hours, but after eight or ten *séances* it persists. After twenty or thirty treatments, Romberg's sign disappears. Then vesical troubles are lessened or removed, also the lightning pains. Sexual impotence gives place to sexual desires and erections. (Experiments by Dr. Onanoff on healthy persons have shown that this method has an exaggerating effect on virility.) The cotton-wool feeling in the feet gives way, more or less, to healthy sensations, and, in general, the whole health improves. Every patient steadily improved, with one exception—a young tabetic of 32, who at first improved, then fell off, then again improved somewhat. But the knee-jerks have not reappeared in any of the patients, after three months' treatment, nor are the pupillary symptoms altered. As to other diseases, a young female with Friedreich's disease was greatly improved by the treatment. In neurasthenic and impotent patients, the sexual functions were reëstablished.

Suspension has also proven beneficial "in[7] Parkinson's disease (paralysis agitans), the rigidity and stiffness having given way to a great degree, the sensations of *chaleur* (flushings) have abated, the sleep is improved, and the gait has become less fatiguing. The tremor, however, remains as persistent as ever."

April 15th, 1889. Bernhardt[8] reported to the Berlin Society for Mental and Nervous Diseases the result of 219 suspensions made in nineteen cases. No unfavorable symptoms observed.

(5) Italics ours.
(6) London Med. Recorder, March 20, 1889.
(7) Krauss, Buffalo Med. Jour., June, 1889.
(8) Centralblatt fur Nervenheilkunde, Psychiatrie, etc., June 1, 1889.

Several patients complained of pain in nuchal muscles. Frequency of pulse unchanged. Traumatic paralysis not observed. In certain cases *decided* improvement occurred in different ways.

1°. Lancinating pains were relieved.

2°. Patients can walk firmer and longer.

3°. Urinating is improved.

4°. Sleep is better.

5°. Don't fall out of bed any more.

6°. Sexual power has been regained.

7°. Many say they feel better, stronger.

Suspension is harmless, yet potent to benefit certain symptoms.

The mental influence is to be considered.

The therapeutic *modus operandi* is as yet undetermined. The physician should always superintend suspension.

Great care is necessary in cases suffering from heart affections.

Eulenberg states that he and Mendel obtained similar results. Forty cases were treated from February 3d to May 10th, 1889; thirty-one were men and nine women. One case of pseudo tabes (woman) hysteria. Marked improvement.

One case of chronic myelitis (woman). No improvement.

Three cases of paralysis agitans. One withdrew from treatment. One no improvement. One walking better. Muscular spasm in arm relieved.

One case, traumatic neurosis (man). No improvement.

Thirty-four cases of tabes. Several cases had sixty suspensions. Some were suspended daily. Of these five withdrew from treatment, six were discharged unrelieved, two were relieved, one after thirty-nine and the other after sixty suspensions.

Still under treatment, twenty-one. Of these four were markedly improved, twelve somewhat relieved and five not affected.

In one case syncope occurred at the commencement of treatment. This is the only case in which an unfavorable effect was observed.

In referring to the treatment of locomotor ataxia and transverse myelitis by suspension, in a clinical lecture delivered at the New York Post-Graduate Medical College, Dr. Chas. L. Dana, Professor of Nervous and Mental Diseases, says: The patient I show you here is the ninth case of locomotor ataxia which I have submitted to this treatment in the past two months. Of these four have had from twelve to sixteen suspensions, and of the five three report decided improvement in the gait and in sensory troubles. I have also employed the treatment in four cases of transverse myelitis. One discontinued treatment after a few trials, and I think it hurt him; two have just been treated only a short time; one has been remarkably benefited as regards strength in the legs and greater freedom of movement.

In certain cases, not recent, of dorsal myelitis in the middle or upper dorsal region, the suspension treatment will, I think, be of much service. But it must be employed carefully. The patients are suspended with about equal support to the head and under the shoulders. After a time, more weight may be thrown on the head.

Dr. R. M. Simpson, of Winnipeg, Canada, reports[9] two cases of tabes as materially benefited by suspension.

Edward Waitzfelder, M. D.[10] Neurologist to the Randall's Island Hospital, New York, says: I began the suspension treatment in the nervous ward of the Randall's Island Hospital with but little hope of benefit, and more from a sense of duty to the unfortunate patients than from any expectation of doing good.

The patient was raised just far enough to let the tips of his toes touch the ground. In some of the cases the loops were put under the axilla; in others they were left off.

CASE I.—M. O'S——, aged thirty-nine. History (of ataxia) for three years. No pains since treatment began. Gait improved. Patient says he feels stronger. After twenty-four suspensions gait very much improved. Ataxia in walking markedly diminished. Can walk almost in a straight line with eyes closed.

CASE II.—Aged thirty-eight. Cerebellar hemorrhage (?) No objective improvement.

---

(9) Canadian Practitioner, June, 1889.

(10) Medical News, June 1, 1889.

CASE III.—J. D——, aged sixty-three. History for eight years. After fifteen suspensions can stand for short time when eyes are closed; gait very much improved; can now walk without a cane; numbness of feet diminished. The feet, which were always cold before, are now warm. Can pass urine easier than before. No improvement in pains after twenty-four suspensions. Patient thinks they are more frequent and severe than before treatment. Gait very much improved; formerly walked with a marked ataxia of feet, bringing down the heel with a severe shock. Now the walk is very slightly characteristic and the "stamp" of the heel very little, and would hardly be noticed. Subjective increase of strength in lower extremities and back. Cramps in legs, which were frequent before, have entirely left him.

CASE IV.—Aged fifty-six. History for nine years. After fifteen suspensions much improved in general condition; walks better; legs feel stronger. Has had no pains at all since suspension was commenced. Urinates with less difficulty and bladder less irritable. Gait much improved; but little "stamp" to the heel. "Can walk a mile now with as little fatigue as a quarter of a mile before."

CASE V.—A. C.——, aged fifty-one. History for three years. Until four days before suspension was commenced, patient was confined to bed for over eight months on account of extreme ataxia of feet and legs. After sixteen suspensions his gait is fairly good, though it still shows some inco-ordination; he can follow a straight line for a short distance; can start walking and stop abruptly without much staggering; can turn about suddenly ("right-about-face") with only a little wavering. Has not been in bed in the day-time since treatment was commenced. Diplopia almost gone; subjective increase of strength in lower extremities.

CASE VI.—C. B.——, aged fifty-three. History for five years. Patient used to sit in the ward all day, very seldom moving from his chair; said he was not able to walk. After fifteen suspensions so much improved that he insisted on having his discharge, saying he was entirely free from pain and could walk as well as he ever could. The improvement was marked from the first suspension.

That there is a direct relation between the effect and cause in these cases there can be no doubt, and that there had been a direct impression produced on the spinal cord, but in what way I am as yet undecided. It is likely that the traction exerted on the spinal nerves in some way brings about a change in the circulation and nutrition of the cord, and the amelioration of the symptoms is due to a lessening of the vascular supply of the cord and its membranes.

On May 1, 1889, D. D. Stewart, M. D.[11] read a paper before the College of Physicians of Philadelphia reporting the treatment of fourteen cases of disease of the spinal cord by suspension, of which cases 1, 2, 3, 4, 6, 7 and 8 were locomotor ataxia (second stage). Case 5 was locomotor ataxia (first stage). Cases 9, 10 and 11 were primary spastic paraplegia. Cases 12 and 13 were combined lateral and posterior sclerosis, and case 14 was one of sub-acute dorso-lumbar meningeo-myelitis.

He closed his paper as follows:

The total number of suspensions made in these cases is upwards of 352. In the tabetics, the symptoms which were first and most strikingly relieved were the ataxia and the lancinating pains; inprovement in these occurred in all, and after a very few suspensions; and that, if any relation existed between the early appearance and extent of the improvement and the duration of the disease, it is a direct one; the more chronic cases being those most promptly and decidedly benefited. The improvement in these two symptoms was so prompt and decided that it may well be considered truly remarkable.

Bladder and rectal difficulty, present in two of the tabetics, and increased sexual desire present in one, have disappeared. Diminution in, or loss of, sexual desire, present in five, has improved in but one. Tactile anæsthesia has much improved in three and slightly in one; not at all in four. A somewhat melancholic condition, present in five of the tabetics, disappeared after a few suspensions. This was not due directly to the suspensions, but

(11) The Medical Report, June 8, 1889.

was rather secondary to the lessening of the pains and ataxia. In none of the eight has the knee-jerk returned, even with reinforcement.

In the three spastic cases, diminution in the paralysis, rigidity, and spasm has been striking in one and slight in another; in the third, no change has occurred in these symptoms.

In one of the ataxic paraplegic cases ataxia and paralysis seemed to be lessened after the second suspension. Unfortunately, this improvement was not maintained, possibly because suspension was not persevered in. The other case writes me that there has been marked improvement in gait and pains.

In the case of myelitis, the improvement in the paraplegia and in the general condition has been decided.

It may be safely asserted, from the favorable results obtained in nearly all of the tabetic cases, that suspension is of great service in locomotor ataxia, but, whether palliative merely or actually curative, the time has not arrived for definite reply. It is certainly of use in lateral sclerosis; and, in myelitis, the decided improvement produced in one case, in which absolute paraplegia had existed over six months, commends its trial in others. As an adjunct to other modes of treatment, its utility is beyond question.

Apropos to a question quite frequently propounded to-day, we make the following extract[12]:

"HOW DOES SUSPENSION ACT IN LOCOMOTOR ATAXY?"

"1°. It has been ascertained that in tabes, posterior spinal meningitis habitually accompanies the pathological changes in the nerve tubes of the posterior columns. The *pia mater* is found congested and thickened at the level of the posterior columns. * * * It appears to me highly probable that part of the influence of suspension, by which the spinal cord is efficiently stretched, is owing to the *breaking down of adhesions from chronic meningitis*, thus allowing freer transmission of nervous influence along the nerve tubes. * * * The neuroglia, from being originally soft and yielding, gradually, as the disease pro-

(12) Julius Althaus, M. D., The Lancet, Apr. 13, 1889.

gresses, loses its cells and nuclei, becomes firm, hard and fibrous, and is liable to cicatricial shrinking. * * * Now it seems to me allowable to assume that by the process of stretching the spinal cord, *the overgrown and unduly hardened neuroglia may be loosened and broken down*, with the effect that those nerve tubes which have to some extent survived the sclerotic process are freed from compression, become better nourished and may thus be enabled to transmit the nervous influence more efficiently than before. Apart from this, however, I have come to the conclusion that suspension has, in a number of cases, a beneficial influence on the *medulla oblongata*, as it stimulates the centers for vaso-motor and cardiac action and for digestion. * * * In a large majority of my cases the appetite and digestion have improved, and mental depression has been lessened or improved.

"The forms of nervous disease, for which my personal experience leads me to think that suspension is applicable, are the following:—1. Locomotor ataxy in the second stage. 2. Paralysis agitans. 3. Spastic spinal paralysis. 4. Amyotrophic lateral sclerosis. 5. Functional nerve prostration, more especially where there is feeble action of the heart, loss of appetite and severe mental depression."

The recent plausible explanation as to the action of suspension in tabes, offered by Althaus, indicates that permanent benefit may result from this method, provided that the further progress of the disease can be arrested by other means, and that it has not advanced to complete destruction of the nerve tubules; and that even where its advance cannot be thus stayed, temporary good may still be accomplished by suspension. Althaus' ingenious explanation applies equally to other degenerative diseases of the cord than tabes, and if it is true of the chronic degenerations, in which the pathological changes originate in the nerve elements and the over-growth of connective tissue is secondary, it is, theoretically, far more so of inflammatory processes which, starting in the connective tissue, cause, primarily, at least, far less damage to the nerve tubes. In chronic inflammations of the cord or its investing membranes, which are stationary and are encountered before the contracting perineural tissue has, by compression, done more than interfere with the nutrition and conductivity of the fibres, not irreparably damaging their structure, the effect of suspension should be curative; for, by stretching the cord and the thickened adherent membranes, the inflammatory tissue pressing upon the axis-cylinders will be loosened and broken down, setting free the latter and permitting them to resume their functions.

All of the suspensions reported by Motchowkowsky, of Odessa; Charcot, of Paris; Abadie, of Paris; Onanoff, Mendel, Barnhart and Eulenberg, of Berlin; Dana, of New York; Simpson, of Canada; Waitzfelder, of New York; Stewart, of Philadelphia; Althaus, of London; Saundby, of Birmingham; and others, have been made by means of the Sayre's suspension apparatus. A feeling that danger lurked in this crude mode of suspension, which is rendered a certainty by the report of a fatal result in *Le Praticien* for May, 1889, led me to contrive the suspension apparatus shown in figures 1 and 2, which was made for me and is for sale by the A. M. Leslie Surgical Instrument Co., of St. Louis.

This device secures (*a*) ready adaptation and facility of application to any sized individual without changing length of straps.—(*b*) Completeness of control of patient.—(*c*) Suspension entirely by either shoulders or head, or by both conjointly and equally, or conjointly yet unequally, to any desired degree, to the fraction of a pound.—(*d*) Exactness as to amount of tractile force exerted at any moment on the neck.

Its use prevents intentional as well as unconscious deception by the patient as to the amount of weight he is supporting by his head, thus placing the operator without the pale of possible mistakes. It affords an accurate basis for dosage of traction and reports of results. In short, it secures perfect precision, which is the essence of science. It will readily be seen that the scales and attached pulleys when detached afford an instrument peculiarly adapted to the accurate stretching of long peripheral nerves, and of reducing luxation. And the surgeon will discover in this invention the most perfect apparatus yet devised for suspending those suffering from spinal diseases and deformities relegated to his domain. And lastly by removing the head-strap and shifting the ropes from the upper to the lower cross-bar and fitting a piece of board into the loops at their lower extremity, a perfect office weighing outfit, much needed by insurance examiners, is secured.

So many perfectly reliable observers working in the interests of science and humanity present such a consension of opinion, regarding the beneficial results to be obtained from suspension in certain diseases of the spinal cord, that I am convinced that benefit will accrue from its use in many cases of tabes dorsalis, Friedereich's disease, neurasthenia, impotence, paralysis agitans, transverse myelitis, spastic spinal paralysis and amyotrophic lateral spinal sclerosis. And I shall also give it a trial in chorea, disseminated sclerosis, hysteria, epilepsy, myoclonus multiplex, myotonia, monospasm, lumbago, sciatica and other allied affections. The *technique* of the operation of suspension as performed by myself, and a report of the results of treatment in fifteen cases, omitted because of lack of space, will appear in the St. Louis *Medical and Surgical Journal* for August, 1889.

NOTE.—Since writing the above I see in the *N. Y. Medical Journal* of May 11, 1889, that Dr. W. A. Hammond has introduced a spring scales into his apparatus.

*St. Louis, June 10, 1889.*

